# Supplementary material for: Learning curve and surgical outcome of robotic assisted colorectal surgery with ERAS program
Source: Sci Rep. 2022 Nov 29;12:20566. doi: 10.1038/s41598-022-24665-w (PMC9709162; doi:10.1038/s41598-022-24665-w)
Supplement: Supplementary file 1 — Supplementary Information 1. [file 41598_2022_24665_MOESM1_ESM.zip › LSC Raw data-Ñ[▒K/113/H_ñw╝╨░O▒Kñσ.pdf]

正式摘要(電子簽章：000000208910032)

W55 / W55 ☐ 英文標題(注意:無英文姓名-請補病人基本檔!)

☐【讀取】憑證

# 臺中榮民總醫院
## 出院病歷摘要

轉入號 : 轉出號 :

[redacted]

| | |
|---|---|
| **(9)**入院日期 | 110/01/20 (2021) 直腸外科 W55 - 272 |
| **(10)**轉科日期 | |
| **(11)**出院日期 | 110/01/26 (2021) 住院天數計 6 日 |

## 診斷

- 入院 :Descending colon adenocarcinoma
  hypertension type 2 diabete mellitus hyperlipidemia
- 出院 :Descending colon adenocarcinoma s/p subtotal colectomy on 2021/1/21
  hypertension type 2 diabete mellitus hyperlipidemia

## 主訴

- pale was noted 4 months ago

## 病史

- This 70 years old female patient has past history of hypertension, type 2 diabete mellitus, hyperlipidemia and operation history of total hysterectomy for uterine myoma about 20 years ago. She recieved regular follow-up in Infection OPD for fever of unknown origin. Anemia was accidentally found in lab data. She was referred to GI OPD after no specific finding in Hema OPD. One huge ulcerative lesion over proximal D-colon was found and adenocarcinoma was proved. Abdominal CT showed regional nodal metastasis but no distal organ metastasis. Thus, she was admitted for surgical treatment.

## 體檢發現

-
  - General appearance :
    - easy looking , conscious clear , without cardiopulmonary distress
    - Vital signs自動帶入date 體溫(BT) 脈搏(P) 呼吸(R) 血壓(BP) 身高(BH) 體重(BW) BMI
      20210120 151cm 63.3Kg 27.8
  - HEENT :
    - Eyes: no anemic conjunctiva, no icteric sclera, normal EOM
    - ENT: no otorrhea, no rhinorrhea, no erythematous mucosa, no injected tonsil, no deformity
  - Neck: supple, freely movable, trachea midline,no deformity, no engorged jugular vein , no palpable thyroid or lymph node enlargement
  - Chest:
    - Inspection: Grossly normal, symmetric and fully expansion, no accessory muscle used, no operation scar, no spider angiomata, no gynecomastia
    - Palpation: no palpable mass or nodule, no subcutaneous emphysema, no tenderness
    - Ausculation: clear breathing sound
  - Heart:
    - Percussion: normal heart size
    - Ausculation: regular heart beat, normal S1, S2, no gallop, no murmur
  - Abdomen:
    - Inspection: in
      - flat contour, symetrically, no scar, no stria, no dilated veins, no rashes
    - Ausculation: normoactive bowel sound, no abdominal bruits
    - palpation: soft, no muscle guarding or rigidity, no local tenderness, no rebounding pain, no palpable mass, no palpable pulsation

- 
    - Percussion: no shifting dulllness, no hepatomegaly, no splenomagaly

- 

| Peripheral pulsation : | CA | BA | RA | FA | PA | PTA | DPA |
|---|---|---|---|---|---|---|---|
| Right side : | ++ | ++ | ++ | ++ | ++ | ++ | ++/+++ |
| Left side : | ++ | ++ | ++ | ++ | ++ | ++ | ++/+++ |

- Genito-anus: no tenderness or palpable mass lesion or rectal shelf, normal external genitalia, normal anal tone, brown stool

## CRS CONDITION

- Colonoscope report: one huge ulcerative lesion over proximal D-colon
- CEA: 1.85 ng/ml
- 
    - Lung

## 手術日期及方法

- 手術日期及方法20210121 1.Laparoscopic converted to conventional subtotal colectomy (due to severe adhesion) with end to end anastomosis, Endo-GIA 60/4.3mm, CDH 29mm, Re-inforcement with silk: nil, air leak test: yes, no leakage.
  2. Vessels ligation of right colic, middle colic artery, left colic and IMA
  3. Cut margin: proximal 25cm, distal 25cm
  4. One 12 Fr.Penrose drain placed in the pelvis

## 住院治療經過

- After admission, operation with subtotal colectomy was done on 1/21 as schedule. After operation, we let her try diet as her tolerance. Penrose drain was removed on POD4. Under the stable condition, she was discharged on 1/26.

## 合併症

- nil

## 一般檢查

- 累積報告 :

| DATE | TIME | ALB | TP | BIL.T | BIL.D | ALKP | AST | ALT | LDH | CHOL | TG | HDL-C | LDL | UA | NH3 | AMY |
|---|---|---|---|---|---|---|---|---|---|---|---|---|---|---|---|---|
| 1100120 | 1921 | 3.7 | 7.0 | 0.43 | 0.09 | 64 | 15 | 12 | 195 | 143 | 136 | | 80 | 6.0 | | |
| 1100121 | 1409 | 2.9 | | 0.3 | | | 14 | 10 | | | | | | | | |

| DATE | TIME | WBC | RBC | HGB | HCT | MCV | PLT | BLST | NEUT | BAND | SEG | LYM | MON | EOS | BAS |
|---|---|---|---|---|---|---|---|---|---|---|---|---|---|---|---|
| 1100120 | 1920 | 10880 | 3.50 | 8.7 | 27.8 | 79.4 | 355 | | 69.6 | | | 20.2 | 7.9 | 2.0 | 0.3 |
| 1100121 | 1404 | 26890 | | 7.8 | | | 310 | | 91.4 | | | 3.5 | 4.8 | 0.1 | 0.2 |
| 1100125 | 0916 | 11690 | | 8.6 | 26.4 | | 292 | | 76.7 | | | 12.1 | 8.3 | 2.7 | 0.2 |

| DATE | TIME | NA | K | CL | CA | BUN | CREAT | Mg | P | CRP | ESR | PCT | LIPASE | LACTATE |
|---|---|---|---|---|---|---|---|---|---|---|---|---|---|---|
| 1100120 | 1921 | 132 | 4.2 | 97 | 8.5 | 12 | 0.83 | | | 0.076 | | | | |
| 1100121 | 1409 | 136 | 3.6 | | | 12 | 0.92 | | | | | | | |
| 1100125 | 0934 | 135 | 3.7 | | | 3 | 0.65 | | | | | | | |

| DATE | TIME | GLUCOSE | PAB | TRF | ZN | CU | KETONE | CK | CKMB | PT-P | PT-C | APTT-P | APTT-C | TRO-T |
|---|---|---|---|---|---|---|---|---|---|---|---|---|---|---|
| 1100120 | 1917 | | | | | | | | | 10.7 | 10.3 | 27.0 | | |
| 1100120 | 1919 | 132 | | | | | | | | | | | | |

## 特殊檢查

## 病理報告

## 其它

## 出院時情況

- 3.改門診治療

## 出院指示

- 

| 藥名 | 劑量 | 劑量單位 | 用法 | 頻次 | 數量 | 數量單位 | 現況 | 天數 | 單位 |
|---|---|---|---|---|---|---|---|---|---|
| Levemir 300U/3ml | 13 | U | SC | QD | 1 | U | TH | 7 | 天 |
| | | | | | | | | | |

| | | | | | | | | | |
|---|---|---|---|---|---|---|---|---|---|
| $Lederscon tab | 1 | TAB | PO | QID | 28 | TAB | TH | 7 | 天 |
| Diovan tab 80mg | 1 | TAB | PO | QD | 7 | TAB | TH | 7 | 天 |
| Acetylcysteine tab | 1 | TAB | PO | QN | 7 | TAB | TH | 7 | 天 |
| JanuMET tab 50/500 | 1 | TAB | PO | BID | 14 | TAB | TH | 7 | 天 |
| 5/20mg Caduet tab | 1 | TAB | PO | QD | 7 | TAB | TH | 7 | 天 |
| Bisco tab 8mg | 1 | TAB | PO | QID | 28 | TAB | TH | 7 | 天 |
| Dobecon tab 100mg | 1 | TAB | PO | BID | 14 | TAB | TH | 7 | 天 |
| Smecta powder 3g | 1 | PKG | PO | QD | 7 | PKG | TH | 7 | 天 |
| Spironolactone-25 | 1 | TAB | PO | BID | 6 | TAB | TH | 3 | 天 |
| $Biofermin tab | 2 | TAB | PO | TID | 42 | TAB | TH | 7 | 天 |

## 出院計劃

主治醫師 2062F 蔣鋒帆 / 住院醫師 2331F 陳凱祥

**正式摘要(電子簽章：000000208910022)**

W55 / W55

doctorRank:B
